# Oxidative Stress and Diseases: Clinical Trials and Approaches

**DOI:** 10.1155/2016/3458276

**Published:** 2016-10-19

**Authors:** Eiichiro Ichiishi, Xiao-Kang Li, Eugenio L. Iorio

**Affiliations:** ^1^Department of Medicine, International University of Health and Welfare Hospital, Tochigi, Japan; ^2^Division of Transplantation Immunology, National Research Institute for Child Health and Development, Tokyo, Japan; ^3^International Observatory of Oxidative Stress, Free Radicals and Antioxidant Systems, Parma, Italy

Due to the fact that oxidative stress may play important roles in human disease development, great many basic and experimental studies have been conducted to clarify the mechanisms that regulate the imbalance between prooxidant and antioxidant systems. Knowledge and understanding of these mechanisms have led to the development of clinical trials and approaches, which have resulted in successful diagnosis and therapies, as well as novel tools to characterize these clinical mechanisms and provide better care to patients.

As to evidence-based clinical applications in humans, significant progress is expected in future medicine. Identifying oxidative stress markers in humans is important, as well as developing strong tools in health promotion and disease prevention for outpatient clinics, bedside monitoring, and home medical care.

In recent years, emerging evidence suggests oxidative stress may play an important role in many skin diseases and skin aging, possibly including atopic dermatitis. The paper by H. Ji and X.-K. Li entitled “Oxidative Stress in Atopic Dermatitis” provides an overview of an update on scientific progress linking oxidative stress to AD and discusses future treatment strategies for better disease control and improved quality of life for atopic dermatitis patients.

There have been intense interest and active researches in the area of dietary antioxidants to develop functional food products. The work by W. Huang et al. entitled “Effect of Blueberry Anthocyanins Malvidin and Glycosides on the Antioxidant Properties in Endothelial Cells” reports that blueberries are a good resource of anthocyanins, which can protect cells from oxidative deterioration, and used as a potential functional food to prevent diseases related to oxidative stress.

Peritonitis, which is the inflammation of the peritoneal tissue, can cause systemic inflammatory response and sepsis. Etanercept is the competitive inhibitor of TNF-*α* which inhibits the binding of TNF-*α* to cell surface receptors and limits its biological activity. In the work by Y. Yildirim et al. entitled “Effect of Intraperitoneal Etanercept on Oxidative Stress in Rats with Peritonitis” the authors showed that the TNF-*α* inhibitor, etanercept, in addition to antibiotics given in the early treatment of peritonitis results in more significant improvement of histopathological and oxidative parameters as compared to antibiotics alone.

The pathogenesis of chronic spontaneous urticaria (CSU) has not been fully understood; nevertheless, significant progress has been achieved in recent years. In the work by F. Dilek et al. entitled “Oxidative Stress in Children with Chronic Spontaneous Urticaria” the authors showed that plasma oxidative stress is increased in children with chronic spontaneous urticaria when compared to healthy subjects, and plasma oxidative stress markers are positively correlated with disease activity.

High levels of circulating low-density lipoprotein (LDL) are a primary initiating event in the development of atherosclerosis. Recently, the antiatherogenic effect of polyphenols has been shown to be exerted via a mechanism unrelated to their antioxidant capacity and to stem from their interaction with specific intracellular or plasma proteins. In their paper “Punicalagin Induces Serum Low-Density Lipoprotein Influx to Macrophages,” D. Atrahimovich et al. demonstrate that, upon binding, punicalagin, the main polyphenol in pomegranate, stimulates LDL influx to macrophages, thus reducing circulating cholesterol levels.

Schizophrenia is a complex, heterogeneous, and severe psychiatric illness that affects about 1% of the population. Exact molecular mechanisms underlying the pathogenesis of this disorder remain to be elucidated. Preclinical and clinical studies of the last decade have highlighted a number of data demonstrating the involvement of oxidative stress in the pathophysiology of psychiatric diseases. In the paper by K. Kriisa et al. entitled “Antipsychotic Treatment Reduces Indices of Oxidative Stress in First-Episode Psychosis Patients,” the authors evaluated the markers of total antioxidative capacity, lipid peroxidation, and protein oxidation and revealed no high-grade oxidative stress in first-episode psychosis patients. Nevertheless, antipsychotic treatment induced a considerable anti-inflammatory effect. Oxidative stress levels were also significantly decreased if compared in first-episode psychosis patients before and after antipsychotic treatment.

Renal transplantation has been considered the best therapeutic option for end stage renal disease. To determine the effect of renal transplantation on the evolution of oxidative DNA status, the work by J. I. Cerrillos-Gutiérrez et al. entitled “The Beneficial Effects of Renal Transplantation on Altered Oxidative Status of ESRD Patients” reports that patients with end stage renal disease have important oxidative damage before RT. The RT significantly reduces oxidative damage and partially regulates the antioxidant enzymes.

Xeroderma pigmentosum group A (XPA) is a genetic disorder in DNA nucleotide excision repair (NER) with severe neurological disorders, in which oxidative stress and disturbed melatonin metabolism may be involved. In the work by R. Miyata et al. entitled “Circadian Rhythms of Oxidative Stress Markers and Melatonin Metabolite in Patients with Xeroderma Pigmentosum Group A” the authors showed that the administration of melatonin has the possibility of ameliorating the augmented oxidative stress in neurodegeneration, especially in the older XPA patients, modulating the melatonin metabolism and the circadian rhythm.

Albumin supplementation of culture media induces sperm capacitation in assisted reproduction technique cycles. Synthetic serum supplementation is clinically used to replace albumin for preventing transmission of infectious agents. However, the effects of synthetic serum supplementation on sperm capacitation have rarely been investigated. In their paper “Effects of Synthetic Serum Supplementation in Sperm Preparation Media on Sperm Capacitation and Function Test Results,” Y.-F. Shih et al. demonstrate that the effects of synthetic serum supplementation on sperm capacitation varied according to the combination of media. These differences may lead to variations in spermatozoon ROS levels, thus affecting sperm function test results.

Molecular hydrogen therapy has been widely concerned and researched. Many animal experiments were carried out in a variety of disease fields. The contribution by Y. Zheng and D. Zhu entitled “Molecular Hydrogen Therapy Ameliorates Organ Damage Induced by Sepsis” provides an overview of molecular hydrogen therapy that can reduce damage of various organ functions from sepsis and improve survival rate. Molecular hydrogen therapy is a prospective method against sepsis.

The positive health benefits stemming from physical activity are well established. In their paper “Effects of Moderate Aerobic Exercise on Cognitive Abilities and Redox State Biomarkers in Older Adults,” A. H. Alghadir et al. demonstrate that moderate aerobic training for 24 weeks has a positive significant effect in improving cognitive functions via modulating redox and inflammatory status of older adults.

Oxidative stress is one of the key mechanisms affecting the outcome throughout the course of organ transplantation. S. Shi and F. Xue in the paper entitled “Current Antioxidant Treatments in Organ Transplantation” provide an overview of emerging antioxidant treatments, targeting donor, graft preservation, and recipient as well.

Kidney ischemia/reperfusion injury emerges in various clinical settings as a great problem complicating the course and outcome. The paper by A. Kezic et al. entitled “Mitochondria-Targeted Antioxidants: Future Perspectives in Kidney Ischemia Reperfusion Injury” provides an overview of the current status of results achieved in numerous studies investigating these novel compounds in ischemia/reperfusion injury which specifically target mitochondria such as MitoQ, Szeto-Schiller (SS) peptides (Bendavia), SkQ1 and SkQR1, and superoxide dismutase mimics.

Cigarette smoking is the most important risk factor for the development or deterioration of Graves' ophthalmopathy. Smoke-induced increased generation of reactive oxygen species may be involved. In the paper by H.-C. Kau et al. entitled “Cigarette Smoke Extract-Induced Oxidative Stress and Fibrosis-Related Genes Expression in Orbital Fibroblasts from Patients with Graves' Ophthalmopathy,” the authors evaluated some clues for the impact of cigarette smoking on Graves' ophthalmopathy and offered a theoretical basis for the potential and rational use of antioxidants in treating Graves' ophthalmopathy.

The molecular mechanisms for hypoxic environment causing the injury of intestinal mucosal barrier (IMB) are widely unknown. To address the issue, Han Chinese from 100 m altitude and Tibetans from high altitude (more than 3650 m) were recruited. The work by K. Li et al. entitled “Genome-Wide Transcriptional Analysis Reveals the Protection against Hypoxia-Induced Oxidative Injury in the Intestine of Tibetans via the Inhibition of GRB2/EGFR/PTPN11 Pathways” reports that the transcriptome analysis showed the protecting functions of IMB patients against hypoxia-induced oxidative injury in the intestine of Tibetans via affecting GRB2/EGFR/PTPN11 pathways.

Mitochondrial superoxide dismutase 2 (SOD2) converts superoxide anions to hydrogen peroxide and oxygen. Human data on SOD2 protein content in chronic kidney disease (CKD) are sparse and mortality data are lacking. In the work by K. Krueger et al. entitled “Lower Superoxide Dismutase 2 (SOD2) Protein Content in Mononuclear Cells Is Associated with Better Survival in Patients with Hemodialysis Therapy” the authors showed that SOD2 protein content declined in CKD until stage 4 while SOD2 gene expression did not. Increased cellular superoxide anion production might affect SOD2 protein content. In advanced CKD (stage 5), SOD2 protein content increased again, but higher than median SOD2 protein content in these patients did not confer a survival benefit.

Overall, several new findings have been presented in this special issue which have further advanced our knowledge in this area. We hope that the readers of this special issue appreciate the progress and the new strategies developed in the field.

